# Current status and emerging role of glutathione in food grade lactic acid bacteria

**DOI:** 10.1186/1475-2859-11-114

**Published:** 2012-08-25

**Authors:** Sarang Dilip Pophaly, Rameshwar Singh, Saurabh Dilip Pophaly, Jai K Kaushik, Sudhir Kumar Tomar

**Affiliations:** 1 Dairy Microbiology Division, National Dairy Research Institute, Karnal, Haryana, India, 132001; 2 BioCOS Life Sciences Pvt. Ltd., Biotech Park, Bangalore, Karnataka, India, 560100; 3 Animal Biotechnology Centre, National Dairy Research Institute, Karnal, Haryana, India, 132001

**Keywords:** Glutathione, Lactic acid bacteria, Stress resistance, Thiols, Probiotics

## Abstract

Lactic acid bacteria (LAB) have taken centre stage in perspectives of modern fermented food industry and probiotic based therapeutics. These bacteria encounter various stress conditions during industrial processing or in the gastrointestinal environment. Such conditions are overcome by complex molecular assemblies capable of synthesizing and/or metabolizing molecules that play a specific role in stress adaptation. Thiols are important class of molecules which contribute towards stress management in cell. Glutathione, a low molecular weight thiol antioxidant distributed widely in eukaryotes and Gram negative organisms, is present sporadically in Gram positive bacteria. However, new insights on its occurrence and role in the latter group are coming to light. Some LAB and closely related Gram positive organisms are proposed to possess glutathione synthesis and/or utilization machinery. Also, supplementation of glutathione in food grade LAB is gaining attention for its role in stress protection and as a nutrient and sulfur source. Owing to the immense benefits of glutathione, its release by probiotic bacteria could also find important applications in health improvement. This review presents our current understanding about the status of glutathione and its role as an exogenously added molecule in food grade LAB and closely related organisms.

## Introduction

Lactic acid bacteria are one of the important groups of microorganisms domesticated for the production of diverse fermented products like fermented milks, cheese, sourdough, sausages, fermented vegetables etc. These bacteria also happen to be the most dominant group in probiotic organisms known for their specific health benefits to humans. Besides, many LAB are also used for the industrial or food grade production of biomolecules like vitamins, exopolysaccharides, polyols etc. Owing to their prolific use in industrial fermentation processes and probiotic applications, these organisms have to negotiate and endure harsh surrounding environments. Stress conditions encountered by LAB in different niches can be broadly classified into two categories viz. technological (oxidative, cold, high osmotic and high temperature conditions) and physiological stress (oxidative, low pH, high bile salts and toxins). Different species of LAB have evolved specialized mechanisms to deal with the normally encountered stress conditions in particular niches [1]. These mechanisms essentially involve intricate maneuvering and interplay of various pathways and biomolecules which support the growth of the organism in their respective transient environment [2]. Thiols, distributed widely in biological systems, are one such important class of compounds engaged in stress protection. Important thiol compounds are glutathione, γ-glutamylcysteine, bacillithiol, mycothiol etc. Glutathione, a tripeptide, is ubiquitous in eukaryotic system, found widely in Gram negative bacteria but was known to be scarcely present in Gram positive bacteria [3,4]. However, new insight into glutathione synthesis and metabolism in the latter group necessitates reconsideration of its status and role in LAB and Gram positive bacteria in general.

**Figure 1:**
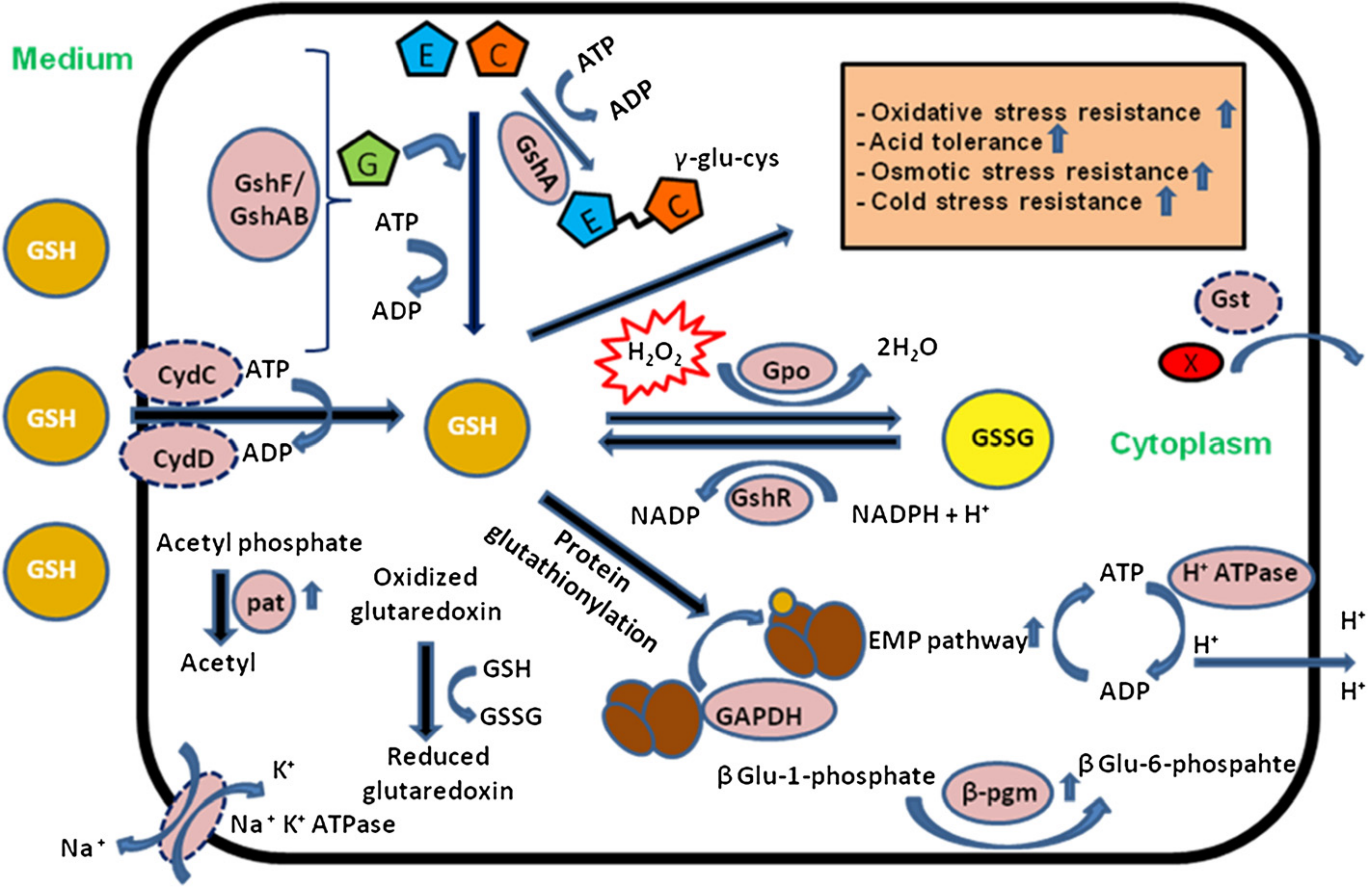
** Schematic diagram showing a putative GSH system and its role in LAB.** A putative GSH system in LAB is illustrated in the figure. Every component may or may not be present in every genus and species of LAB. Genes or proteins whose activity is not yet established in LAB are shown with dotted circle. Glutathione (GSH) is made-up of three amino acids viz. glutamic acid (E), cysteine (C) and glycine (G). The enzymes of glutathione system are γ-glutamylcystiene synthetase (GshA), glutathione synthetase (GshB), glutathione bifunctional fusion protein (GshAB/GshF), glutathione reductase (GshR/Gor), and glutathione peroxidase (Gpo). GshA catalyzes the formation of γ-glutamylcysteine from glutamic acid and cysteine. Some LAB have only GshA homologs making γ-glutamylcysteine as the major thiol. This molecule also serves as antioxidant in some species. The classical two step biosynthesis of glutathione is absent and it is carried by bifunctional fusion protein GshF in some LAB like *S. thermophilus*. Some other LAB also have this fusion protein. Besides its possible *de-novo* synthesis, GSH is also imported from the medium possibly by CydDC, a heterodimeric ATP-binding cassette type transporter. Gpo and GshR are the two main enzymes involved in metabolism of glutathione. Former catalyzes the conversion of reduced glutathione (GSH) to oxidized form (GSSG) and the latter enzyme regenerates the reduced form. Glutathione-S-transferases (GSTs) are a class of enzymes which are involved in cellular detoxification of xenobiotics (X) using reduced glutathione. The exact cellular role of GST in LAB is not yet established. GSH also carries glutathionylation of key proteins of EMP pathway (e.g. GAPDH) and helps to maintain ATP production at required levels during stress conditions. GSH supplementation upregulates activity of enzymes like β-phophoglucomutase (β-Pgm), phosphate acetyltransferase (Pat) etc. during stress conditions [81]. Both synthesized as well as imported GSH is involved in protection of cells from various stress conditions.

Glutathione (GSH) is made-up of three amino acids viz. glutamate, cysteine and glycine. The primary enzymes/genes of glutathione system (Figure 1) are γ-glutamylcystiene synthetase (*gshA*), glutathione synthetase (*gshB*), glutathione reductase (*gshR/gor*), and glutathione peroxidase (*gpo*). The biosynthesis of GSH involves formation of a peptide bond between glutamate and cysteine catalyzed by γ-glutamylcysteine synthetase (GshA) and subsequent formation of a peptide bond between γ Glu-Cys and glycine catalyzed by glutathione synthetase (GshB) [3]. Alternately, some Gram positive bacteria have evolved a single multidomain fusion protein (GshF) which catalyzes both the reactions for synthesis [5,6]. Many organisms can transport glutathione from the medium and utilize it for various cellular reactions. Glutathione transport in prokaryotes is known to be carried by CydDC, a heterodimeric (consisting of two subunits CydC and CydD), ATP-binding cassette type transporter [7]. It contributes to the reducing environment in cell by its ability to transport glutathione and cysteine. Glutathione peroxidase and glutathione reductase are the two main enzymes involved in metabolism of glutathione. Former catalyzes the conversion of reduced glutathione (GSH) to its oxidized form (GSSG) and the latter enzyme regenerates the reduced form. Glutathione degradation is carried by γ-glutamyltranspeptidase (Ggt) which helps in recycling of the constituent amino acids. Additionally, many glutathione dependent proteins found in prokaryotes use the molecule to carry out diverse reactions [8] e.g. glutathione-S-transferase (GST) is a superfamily of enzymes which use GSH to conjugate and detoxify certain xenobiotic compounds. GSH also reduces glutaredoxins (small redox enzymes) which are oxidized by different substrates. 

Glutathione has diverse roles in biological systems for its antioxidative, immune boosting and cellular detoxifying activities [9]. It helps to maintain the intracellular redox homeostasis to protect the cells against oxidative damage. Most of the biological functions of glutathione are mediated by the conversion of reduced glutathione (GSH) to its oxidized form (GSSG) by the enzyme glutathione peroxidase and transforming back to GSH by glutathione reductase, a mechanism which maintains its cellular forms and levels [10]. The ratio GSH/GSSG which works as a cellular redox switch determines the oxidative status of the cells and is delicately maintained by the activity of these two enzymes [11].

The roles of glutathione in Gram negative bacteria have been extensively reviewed [12,13]. However it requires to be detailed in Gram positive bacteria. Here, we review the currently available information on GSH system in food grade LAB supported by genomic analysis for distribution of GSH system genes in their available whole genome sequences. Whole genome sequencing (WGS) has opened new avenues for discovery of molecules and associated pathways e.g. glutathione S-transferase gene was not known to exist in lactobacilli till the WGS of *Lactobacillus casei* Zhang was achieved [14]. WGS also helps to corroborate the biochemical evidence for synthesis/transport of metabolites already known to exist in cell. Further, the role of GSH supplementation in stress protection as well as technological and health implications of microbial GSH metabolism are also discussed.

## Screening of selected LAB genomes for GSH system genes

We looked for eight major genes involved in glutathione synthesis, transport and metabolism in fully sequenced genomes of selected food grade LAB and other organisms of probiotic and dairy importance namely bifidobacteria and propionibacteria. The search was carried out in two different ways; firstly based on the keyword search of annotated protein entries and secondly based on the sequence similarity determined by Reverse Position Specific (RPS) BLAST.

Proteomes of the completely sequenced organisms mentioned above were downloaded from NCBI [15]. Genes annotated to be involved in GSH synthesis and metabolism were searched in Uniprot [16] advanced search using keyword “glutathione” and the organism “taxonomy id”. For glutathione transport genes, following keywords were used “cydD”,“cydC”,“cydDC” and “cydCD”, with the organism taxonomy id. The resulting records were manually filtered to find genes annotated in glutathione synthesis, transport and utilization.

For listing of glutathione system genes based on sequence analysis, RPS-Blast with NCBI Protein clusters database (ProtClustDB) [17] was used. File containing position specific scoring matrices (pssms) of the prokaryotic protein clusters was downloaded from the ProtClustDB. Following protein clusters were manually identified to be involved in GSH synthesis and metabolism viz. GshA (PRK02107, PRK13516, PRK13518, PRK13517, PRK13515), GshB (PRK12458, PRK05246), GshF (PRK02471), Gpo (PRK10606), GST (PRK11752, PRK10357, PRK10542), Ggt (PRK09615), CydC (PRK11160) and CydD (PRK11174). RPS-BLAST (with e-value cutoff of 1e^-5^) was then used to search the individual LAB proteomes against all the pssms from the downloaded file. A protein was assigned to the glutathione system if any one of the above mentioned clusters was the first hit in RPS-BLAST results of that protein against a database of all downloaded PSSMs. For example, GshB has two protein clusters associated with it (PRK12458 and PRK05246), so if the first hit from RPS-Blast for a protein was any one of these clusters, the protein was assumed to be GshB. Since in some cases *gshA* and *gshB* fuse to form a single gene *gshF* and sequence divergence is very high for both GshA and GshB [3], the results of GshA, GshB and GshF were manually checked for the position of alignment in the gene and length of the hit. Hits with protein clusters of GshA and more than 650 amino acids were assumed to be GshF. The results are tabulated in the Additional file 1 and are discussed below with reference to available literature on GSH system in LAB.

## Distribution of glutathione in selected LAB and closely related Gram positive bacteria

Glutathione is ubiquitously found in eukaryotes and is the major indigenous antioxidant in higher animals [3], whereas in prokaryotes it is much widely distributed in Gram negative bacteria [4] although, some members of Gram positive bacteria are suggested either to synthesize or import it from the medium. Fahey and coworkers [4] reported that *Streptococcus lactis* (now *Lactococcus lactis*) produces GSH when grown in trypticase soya broth [Table 1. This proposition was based on the higher GSH content found in cell lysate than the medium and was supported initially from the findings of Fernandes and Steele [18] who reported synthesis of GSH by *Lc. lactis* subsp. *cremoris* Z8 from precursor amino acids supplemented in milk. However, the argument was refuted for the strain Z8 when grown in a chemically defined medium (CDM) and it was found only to accumulate GSH from the medium [19]. Moreover, no GSH could be observed in 21 strains of lactococci when grown in CDM further substantiating the absence of GSH synthesis in the genus [20]. However, lack of genetic studies at that stage prevented any concrete evidence in support for presence/absence of *de-novo* glutathione synthesis in *Lactococcus* spp. GSH accumulation was more strongly observed in the strains of *Lc. lactis* subsp. *cremoris* but was absent in most of the *Lc. lactis* subsp. *lactis* and *Lc. lactis* subsp. *lactis* biovar *diacetylactis* strains [18,20]. Recently, Amaretti et. al. [21] also observed high GSH accumulation by a strain of *Lc. lactis* subsp. *cremoris*. Moreover, glutathione reductase activity was in general higher for *Lc. lactis* subsp. *lactis* than *Lc. lactis* subsp. *cremoris* and was not related to the intracellular GSH content [20]. Glutathione estimation in probiotic strains of lactococci by Capillary Electrophoresis–LIF was reported [22] and it is very likely that the observed glutathione levels are the result of import from the medium (here trypticase soya yeast extract medium). Our genomic analysis [see Additional file 1 revealed the presence of putative transport gene *cydDC* as well as *gpo* and *gshR* genes but no homolog of *gshA* or *gshB* in *Lactococcus* spp. could be detected corroborating the studies showing absence of GSH synthesis in lactococci but possessing the ability to import and regenerate reduced glutathione. *Lc. lactis* which is used as a model organism for food grade production of various molecules was employed for GSH production by genetically engineering *gshA* and *gshB* genes from *Escherichia coli*[23], wherein, a very high concentration of GSH (358 nmol/mg protein) could be achieved by supplementing the medium with precursor amino acids. *Lc. lactis* could also serve host to human glutathione transferase enzyme as means of food grade expression of the latter [24]. 

**Table 1:** Glutathione concentration in food grade LAB and some related Gram positive bacteria

**Organism**	**Possible/ Proposed mode**	**Glutathione concentration**	**Medium**	**Reference**
*S. thermophilus* ATCC 19258	Synthesis	5.7 μmol/g dry weight	Trypticase soy broth	[28]
*S. thermophilus* S6	ND^*^	43.5 nmol/mg	Elliker broth	[18]
*S. thermophilus* ST2	ND^*^	39.2 nmol/mg	Elliker broth	
*S. thermophilus* MB410	ND^*^	10.3 nmol/mg	M-17 broth	[21]
*S. thermophilus* MB426	ND^*^	19.4 nmol/mg	M-17 broth	
*S. agalactiae* ATCC 12927	Synthesis	11 μmol/g dry weight	Trypticase soy broth	[28]
*S. agalactiae* 2603 V/R	Synthesis	327 nmol/mg protein	CDM^***^	[6]
*E. faecalis* ATCC 29212	Synthesis	1.8 μmol/g dry weight	Todd Hewitt medium^***^	[28]
*E. faecalis* JH2-2	Synthesis	5.1 μmol/g dry weight	Trypticase soy broth	
*E. faecium* ATCC 6569	ND^*^	1.6 μmol/g dry weight	Trypticase soy broth	
*E. faecalis*	Synthesis	78 nmol/mg protein	CDM^***^	[32]
*E. faecium*	Synthesis	189 nmol/mg protein	CDM^***^	
*Lc. lactis* subsp. *cremoris* ATCC 19257	ND^*^	26.3 nmol/mg	M-17 broth	[21]
*Lc. lactis* subsp. *diacetylactis* MB447	ND^*^	10.5 nmol/mg	M-17 broth	
*Lc. lactis*	ND^*^	0.358 μg/mg protein	Trypticase soy broth	[22]
*Lc. lactis* ssp. *cremoris* Z8	Synthesis^?^	51.4 nm/mg	Elliker broth	[18]
*Leu. mesenteroides* ssp*. cremoris* CAFT9	ND^*^	12.6 nmol/mg	APT	
*Leu. mesenteroides* ssp*. cremoris* ATCC 19254	ND^*^	11.4 nmol/mg	MRS	
*Lc. lactis* ssp. *cremoris* Z8	Synthesis^?^	15 nmol/mg	Milk	
*Lc. lactis*	Synthesis^?^	4.6 μmol/g	Trypticase soy broth	[4]
*Lc. lactis* (genetically engineered strain)	Synthesis	358 nmol/mg	CDM^***^	[23]
*Lc. lactis* subsp*. cremoris* SK 11	Import	10.56 nmol/mg protein	M17 broth	[20]
*Lc. lactis* subsp*. lactis* NIZO B89	Import	4.95 nmol/mg protein	M17 broth	
*Lc. lactis* subsp*. lactis* NIZO B93	Import	9.13 nmol/mg protein	M17 broth	
*Lb. helveticus* CNRZ 32	ND^*^	6.2 nm/mg	APT broth	[18]
*Lb. casei*	Import	0.05 μmol/g	Lactobacillus broth	[4]
*Lb. casei* HY 2782	ND^*^	25.15 μmol/g	MRS broth	[34]
*Lb. acidophilus* DSMZ 23033	ND^*^	4.5 nmol/mg	MRS broth	[21]
*Lb. acidophilus*	ND^*^	0.14 nmol/mg protein	MRS broth	[41]
*Lb. salivarius*	ND^*^	0.11 nmol/mg protein	MRS broth	
*Lb. casei* W56	ND^*^	0.09 nmol/mg protein	MRS broth	
*Lb. rhamnosus*	ND^*^	~ 14 μmol/g	MRS broth	[34]
*Lb. plantarum*	ND^*^	~ 14 μmol/g	MRS broth	[34]
*Lb. plantarum* LP1	ND^*^	2.7 nmol/mg	MRS broth	[21]
*Lb. salivarius*	Import/ synthesis^?^	~0.5 nmol/mg protein	MRS broth^**^	[40]
*Lb. salivarius*	Import	~22 μmol/10^12^ cells	BHI broth	[111]
*Lb. fermentum* ME-3	Import/ synthesis^?^	20 nmol/mg protein	Milk	[36]
*Lb. fermentum* ME-3	Import/ synthesis^?^	9.95 μg/ml	MRS broth	[37]
*Lb. fermentum* 5716	ND^*^	1.4 mM/ ml media	MRS broth	[39]
*Lb. reuteri* ATCC 23272	Import	~20 mg/g dw	MRS broth^**^	[43]
*Lb. reuteri* ATCC 23272	Import	~15 mg/g dw	MRS broth	
*B. adolescentis* MB238	ND^*^	0.238 μg/mg	MRS broth	[22]
*B. breve* MB233	ND^*^	0.258 μg/mg	MRS broth	
*B. longum* MB243	ND^*^	0.333 μg/mg	MRS broth	
*B. adolescentis* B660	ND^*^	0.288 μg/mg	MRS broth	
*B. bifidum* W23	ND^*^	0.37 nm/ mg	MRS broth	[41]
*B. animalis* subsp. *lactis* DSMZ 23032	ND^*^	30.3 nmol/mg	MRS broth	[21]

^*^not defined.

^**^ GSH supplemented to medium.

^***^Medium devoid of glutathione (either by treating with γ-glutamyl transpeptidase or using chemically defined medium).

^?^ Contradictory report with respect to present available information.

The genus *Streptococcus* consists of some prominent members in which GSH synthesis has either been presumed or experimentally verified. *Streptococcus agalactiae* (a bovine pathogen) was found to produce GSH in trypticase soya broth [4] and also in CDM [6]. *S. agalactiae*[6] is one of the two (other being *Listeria monocytogenes*[5]) organisms first reported to synthesize GSH by a multidomain bifunctional fusion protein (GshF or GshAB) in which the N-terminal domain is responsible for γ-glutamylcysteine synthetase activity and the C-terminal domain carries glutathione synthetase activity. The presence of similar putative fusion proteins was latter suggested in *Streptococcus thermophilus, Streptococcus suis, Streptococcus sanguinis, Streptococcus mutans, Enterococcus faecalis* and *Enterococcus faecium*[6,25]. Recently, GshF fusion protein from *S. thermophilus* was cloned and expressed in tobacco plant (*Nicotiana tabacum*) [26] and *Escherichia coli*[27] to achieve high levels of GSH production. The presence of an active bifunctional fusion protein, high GSH content in the cells grown in Elliker broth [18], M-17 broth [21] as well as in trypticase soya broth [28] and an active GshR (Gor) enzyme [29] indicate towards a functional GSH system existing in *S. thermophilus*. Interestingly, *gshF* gene in *S. thermophilus* is shown to be insensitive to feedback inhibition by GSH allowing high cellular accumulation levels, a property which can be used for the production of food grade glutathione. GshR activity (besides other antioxidative enzymes) helps *S. thermophilus* to adapt several treatments during industrial processing [30]. Similarly, *E. faecalis* and *E. faecium* was also reported to produce high GSH in rich medium [28,31] as well as in CDM [32] and is also able to regenerate the reduced form by glutathione reductase (GshR) activity under oxidative stress [33]. Our analysis showed the presence of transport protein CydDC as well as glutathione biosynthesis fusion protein GshF in *E. faecalis* genome.

Glutathione synthesis, transport and metabolism in *Lactobacillus* genus have been studied for some species but most are yet to be explored. Among lactobacilli, GshF homologs could be detected in *Lb .casei, Lb. rhamnosus, Lb. plantarum, Lb. sakei* and *Lb. ruminis* [see Additional file 1. GSH was found present in the cell lysate of *Lb. casei* but that was attributed to its high concentration in the growth medium and *de-novo* synthesis could not be established [4]. Cellular GSH concentration in *Lb. casei* HY 2782 reached at the highest level after 24 h of growth and tended to decrease thereafter [34]. Highest concentration was achieved in de Man Rogosa Sharpe (MRS) broth as compared to tryptone phytone yeast extract (TPY) and bromocresol purple dextrose (BCP) broth. Thioredoxin-thioredoxin reductase system in *Lb. casei* Shirota is the dominant thiol/disulphide redox system and the *trxA1trxA2* &*trxB* mutants of the strain have severely damaged growth rate which is restored after addition of glutathione in the medium [35]. Externally added GSH thus, may activate a secondary redox system of GSH-glutaredoxin and might indirectly suggest the absence of GSH synthesis to supplement such reactions. This is also confirmed by the presence of putative glutathione transport protein CydDC in *Lb. casei* genome [Additional file 1. Thus, presently *Lb. casei* appears to have the ability to import GSH from the medium with no evidence of synthesis, although a putative fusion protein is found in the genomes. It also has *gshR* and *gpo* homologs suggesting the ability to use the imported glutathione for redox reactions. *Lb. rhamnosus* CU01 was reported to have ~ 14 μmol/g GSH when grown in MRS broth largely expected to be imported from the medium [34] but it does possess a *gshF* homolog. A thorough screening of *Lb. casei* and *Lb. rhamnosus* strains for GSH synthesis in chemically defined medium and genetic analysis of the fusion protein is needed to demonstrate the absence or presence of an active GSH system.

Many workers have suggested the ability of *Lb. fermentum* strains to synthesis GSH based on phenotypic data. *Lb. fermentum* ME-3, a well established and widely studied probiotic strain, was recently reported to have a complete glutathione system characterized by glutathione synthesis, uptake and redox turnover [36]. This organism was isolated from the faeces of an Estonian child [37,38] suggesting that many gut microorganisms may be having glutathione system with vital functionalities for the host. Moreover, glutathione content in *Lb. fermentum* ME-3 was higher when grown in milk as compared to MRS broth, since milk contains all the three necessary precursor amino acids for GSH synthesis and is also a natural growth medium for LAB. *Lb. fermentum* 5716, another probiotic strain was found to be a prolific GSH as well as γ-glutamylcysteine producer [39]. Similarly, GSH synthesis is being presumptively shown in *Lb. salivarius*[40,41]. However, the genetic elements associated with the GSH system in *Lb. fermentum* and *Lb. salivarius* have not been investigated yet. In our analysis, *Lb. fermentum* and *Lb. salivarius* genomes were neither found to possess *gshF* nor *gshB* homolog but only *gshA*. This presents a complex case to explain GSH synthesis by the organism more so by the fact that genomes of *Lb. reuteri*, an organism phylogenetically close to *Lb. fermentum*, also show presence of only *gshA* and absence of both *gshF* and *gshB.* However, no glutathione synthesis has been reported for *Lb. reuteri* and γ-glutamylcysteine (product of *gshA*) is known as the major thiol in the species [42]. *Lb. reuteri* however, has the ability to import GSH [43] from the medium which is also supported by the presence of the putative transport protein [see Additional file 1. This protein, also present in *Lb. fermentum* and *Lb. salivarius* genomes could be implicated in GSH import from the medium. Thus, at present GSH synthesis in both of these organisms is disputable and a thorough investigation using chemically defined medium and expression studies is required to establish the status of GSH system in *Lb. fermentum* and *Lb. salivarius*.

*Lb. plantarum* possesses a putative homolog of bifunctional fusion protein *gshF*[6,25], however, GSH synthesis in this organism has not been reported but it may uptake GSH from the medium [21,34]. Recently, Ge and workers [44] cloned the *gshF* gene from *Lb. plantarum* into *Pichia pastoris* but no improvement in GSH production could be obtained and thus the activity of this enzyme in *Lb. plantarum* is not ascertained till date. Interestingly, *Lb. plantarum* genome contains both *gshA* and *gshF* homologs. Moreover, active thioredoxin system has been reported in *Lb. plantarum*[45] to counteract oxidative stress but a redundant functional glutathione system can exist as it contains both the required genes for redox turnover reaction using glutathione.

Very few reports on presence of glutathione are available for *Lb. acidophilusLb. helveticus* and *Lb. delbrueckii* subsp. *bulgaricus*. Low level of GSH accumulation is observed in *Lb. helveticus*[34] and *Lb. acidophilus*[21,34]. Our genomic analysis showed a near absence of GSH system in these organisms [Additional file 1. A putative *gshF* fusion protein homolog was also observed in *Lb. sakei* (a meat borne LAB) and *Lb. ruminis*. *Lb. sakei* exhibits a wide intraspecies variation in response to oxidative stress and a redundant putative glutathione-glutaredoxin system may be credited to the varied oxidative diversity of the strains [46]. GshF in *Lb. ruminis* (uniprot id: G2SQ55; gene name: LRC_13280) is one of the several specific proteins which is absent in the *Lb. salivarius* (both belonging to the same clade) as shown by comparative genomics of the two species [47]. *Lb. buchneri*, which is used in silage fermentation, is having four copies of *gshA* as found by RPS-Blast in our study but they are possibly misannotated as *gshB* in Uniprot.

Leuconostocs, another important member of LAB, are widely used in fermented vegetables like sauerkraut, kimchii etc. Glutathione was found in *Leu. mesenteroides* cells grown in APT and MRS medium [18]. But, later it was reported that *Leuconostoc* spp. lacks *gshB* (and also *gshF*), thus making it incapable of GSH synthesis. Instead, the intermediate compound γ-glutamylcysteine was present in large amount and higher expression (of *gshA*) was reported after peroxide treatment making this molecule the major thiol in *Leuconostoc* spp. [25]. Although, γ-glutamylcysteine is the dominant thiol, all the *Leuconostoc* species show presence of *gpo* and *gshR* [Additional file 1 thus, import of GSH to mount an antioxidative response based on the two enzymes makes sense. This import could be carried out by the putative CydDC protein found in whole genome sequences of leuconostocs [Additional file 1.

*Oenococcus oenii* is a LAB used in wine fermentation. It possesses both g*shR* and *gpo* homologs but synthesis related genes were found absent, thus justifying the presence of the putative transport protein CydDC [Additional file 1. Glutathione has been considered as one of the hallmark molecules of aerobic metabolism and its detection in obligate anaerobes like bifidobacteria [22,41] is intriguing which needs to be investigated. It is not clear whether the observed content is due to import from the medium or synthesis *per se* is carried out by the strains. However, our analysis showed complete absence of the glutathione system in bifidobacteria. In a recent work [8] it was suggested that many organisms which are having GSH dependent proteins lack *gshA* but still are able to synthesize GSH through a proposed alternate route involving proline biosynthetic pathway. However, in case of LAB, only homologs of *gshA* or *gshF* are present and *gshB* is absent throughout. This scenario supports the hypothesis that LAB which if at all, synthesize glutathione are able to do so by the glutathione biosynthesis bifunctional fusion protein. In LAB and closely related organisms, glutathione synthesis is not indispensible for survival but many LAB may either have synthesis, truncated synthesis, import and/or utilization system for glutathione, with inherent species and strain level variations.

### Distribution of glutathione-S-transferase

Glutathione S-transferases (GSTs) are the part of a superfamily of enzymes that play a key role in cellular detoxification and xenobiotic degradation. GSTs from Gram positive bacteria are grouped into four different classes viz. Beta class, Fosfomycin resistance proteins, Xi class and Ure2 proteins [48]. Till now, in bacterial cultures of dairy origin, only *S. thermophilus*[48], *Lb. casei*[14] and *Propionibacterium freudenreichii*[49] are reported to have glutathione-S-transferase orthologs. *S. thermophilus* GST have been annotated as Ure2 protein but no class has been assigned for the latter two species. *Pr. shermanii* was shown to have antimutagenic properties against 4-nitroquinoline-1-oxide (4NQO) which could be linked to the concomitantly enhanced GST activity as a result of exposure to the mutagen [50]. GSTs are known to degrade biphenyl, but no specific activity of GST in *Lb. casei* could be linked to biphenyl degradation [14] and thus its role still remains obscure.

## Role of glutathione in food grade LAB

Glutathione in Gram negative bacteria is a well established molecule offering resistance in conditions such as oxidative stress [51], radiation stress [52], methylglyoxal resistance [53], osmotic stress [54], chlorine resistance [55] and heavy metal resistance [56]. Glutathione system also accomplices in virulence of certain pathogens [57] by assisting their survival in inflammatory tissues laden with oxidative stress. With its discovery in Gram positive bacteria, new claims on its role in stress resistance of these organisms are coming to light (see figure 1). In recent years, exogenously supplied GSH has been focused on as an important molecule in stress protection and growth promotion of several LAB. Some of the key applications of GSH in LAB and their proposed mechanisms are discussed here.

### Oxidative stress

Oxidative stress is a result of elevated exposure of cells to reactive oxygen species (ROS) e.g. superoxide anions, hydrogen peroxide, hydroxyl radicals and hydroperoxides. LAB encounters oxidative stress during GI transit, various industrial treatments and during co-culturing in fermented foods. Bacteria deploy specialized mechanisms to deal with the oxidative stress [58] which include enzymes like SOD, thioredoxin reductase system, glutathione-glutaredoxin system and NADH oxidase/ NADH peroxidase system. Thioredoxins are small redox proteins which act as antioxidants by reducing other proteins via cysteine thiol-disulphide exchange reactions and themselves are reduced by thioredoxin reductase. Thioredoxins are important for antioxidative response in *Lb. casei*[35] and *Lb. plantarum*[45] but not for *Lc. lactis*[59]. *Lb. fermentum*[60] and *Lb. reuteri*[42] are more dependent upon cysteine/cystine uptake for oxidative stress protection. Low molecular weight (LWT) thiols such as glutathione and cysteine are the major contributors of redox potential changes during stress conditions in bacteria [61]. Thiol groups displayed on the cell surface proteins have also been shown to maintain a reducing microenvironment and help in the oxidative stress protection of *Lc. lactis*[62]. Glutathione is considered as one of the important molecules in oxidative stress protection in model organisms like *S. cerevisiae*[63] and *E. coli*[51]. This major property of glutathione has also now been proved in LAB and is covered below.

Glutathione as a reducing agent in cytoplasm is important in imparting antioxidative properties to the bacteria. It is able to defend cells against oxidative radicals which can severely compromise the survival and performance of the culture. In a study, antioxidative activity of the cell free extract of 11 strains of lactobacilli was found directly related to the cellular reduced glutathione level [34]. Thus, both GSH accumulation and synthesis could be correlated with the antioxidative potential of LAB and its ability to grow in aerobic environment, although other factors are also involved. Aerobically grown cells of *Lc. lactis* subsp. *cremoris* SK11 showed 30% higher GSH accumulation and 5 fold higher glutathione peroxidase activity than anaerobically grown cells which provides a primary evidence for the role of GSH in oxygen tolerance of LAB. *Lc. lactis* subsp. *cremoris* SK11 accumulates GSH and offers increased protection against H_2_O_2_ to stationary phase cells [20] possibly by its ability to mount a GSH-reductase-peroxidase antioxidative response to peroxide induced oxidative stress. These results are consistent with the reports on cellular protection afforded by exogenously supplied GSH in *Haemophilus influenza*[64] and *S. mutans*[65], both of which also lack GSH biosynthesis. Genetic engineering for introducing glutathione synthesis ability in *Lc. lactis* subsp. *cremoris* NZ9000, which is incapable of synthesis as well as uptake of glutathione, resulted in an increased resistance to the oxidative stress induced by H_2_O_2_ and menadione [66]. This finding is in line with the improved tolerance achieved by engineering GSH biosynthesis even in the obligate anaerobe *Clostridium acetobutylicum* against aeration and butanol challenge [67].

Glutathione reductase activity regenerates GSH from GSSG, thus helps to maintain a reduced microenvironment and ensures substrate availability for glutathione peroxidase. Glutathione peroxidase on the other hand utilizes GSH to scavenge reactive oxygen spices (ROS) and relates to the high antioxidative ability of the cells as reported for *Lb. brevis* KCTC 3498 [68]. GshR activity in *Lb. sanfranciscensis* helps to maintain thiol levels in wheat sourdough and have important technological implications for sourdough quality. *Lb. sanfrancisensis gshR* mutants are not able to maintain thiol levels in wheat sourdough, result in a loss of oxygen tolerance and become more sensitive to superoxide generating agent methyl viologen (paraquat) [69]. At elevated oxidative exposure, glutathione reductase activity was reported to be enhanced in *S. thermophilus*[29], *E. faecalis*[33], *Lb. acidophilus* NCFM [70] and *Lc. lactis* subsp. *cremoris* NZ9000 [66] indicating the clear role of this enzyme in oxidative stress protection mechanism in LAB.

GSH may also be helpful in overcoming secondary oxidative stress accompanied with several treatments and conditions. Iron supplementation in the medium presents *E. faecalis* cells with oxidative stress with a consequential decrease in cellular glutathione content [31]. Profiling of genes overexpressed in *Lb. plantarum* WCFS1 after bile exposure showed an increased expression of glutathione reductase suggestively to overcome oxidative stress accompanied with the bile salts [71]. In another report, Gpo activity was enhanced in *Lc. lactis* to counter the secondary oxidative stress induced by isoleucine starvation [72]. Presence of glutathione and GshR-Gpo couple is not indispensible for survival of LAB under oxidative stress but may work as an auxiliary system, apparently because of the other primary antioxidative systems active within the cells.

### Acid stress

LAB encounter acidic conditions in the medium as a result of their own acid generation during fermentation and in the gastrointestinal environment where these bacteria usually thrive. Glutathione has been earlier reported to protect *E. coli*[73] and *Rhizobium tropici*[74] cells from acid challenge by regulation of gated potassium export channels KefB and KefC, restricting transport of K^+^ ions and thus maintaining the cytoplasmic pH. Supplementation of GSH has been shown to protect *Lb. salivarius*[40], *Lc. lactis* subsp. *cremoris*[75] and *Leu. mesenteroides*[76] from varying degree of acid challenge. Regulation of potassium export channels by GSH is not yet proven in LAB and the mechanism for protection is suggested to be either sacrificial action of GSH which prevents rapid fall of intracellular pH or glutathionylation of glyceraldehyde 3-phosphate dehydrogenase (GAPDH) enzyme which helps to sustain glycolysis at the required level. The latter mechanism is also supported by the finding that growth rate was enhanced post GSH supplementation in *Lb. salivarius* cells under acid stress [40]. Synthesized GSH also offered protection against acid stress in genetically engineered strain of *Lc. lactis* NZ9000 [75]. Glutathione reductase activity maintains the GSH concentration in cells and thus is expectedly high for *Lc. lactis* IL1403 [77] and *S. pneumoniae*[78] in the face of acid challenge. Improved acid tolerance in LAB by GSH could be harnessed for increasing their survival under acidic conditions in both vat and gut.

### Cold stress

LAB used in food fermentation processes have their optimum growth temperature in mesophilic or thermophilic range and thus preservation methods essentially involving freezing, low temperature storage or freeze drying impart a certain degree of cold stress to the cells. Industrial performance of the starters is affected vastly by their ability to resist such conditions and maintain high viability [1]. It is suggested that the cold treatments given to *E. coli* cells are physiologically manifested in the form of oxidative stress leading to enhanced expression of Mn-SOD and catalase along with decrease in the intracellular glutathione and GSH/GSSG ratio [79]. Glutathione level decreases during cold stress conditions and thus supplementation of GSH to the medium or native GSH synthesis by bacterial cells could replenish this loss and may help to cope up with such conditions. Survival rates after freeze drying of *Lb. sanfranciscensis* were found to be several folds higher for cells supplemented with GSH as compared to cysteine supplemented cells and control culture [80]. A similar pattern of cellular protection could be observed upon freeze thawing and cold treatment at 4°C. This protective effect of supplemented glutathione is attributed to the prevention of membrane fatty acid oxidation, maintenance of average chain length of fatty acids [80], sustainment of high metabolic activity and protection against secondary stress conditions generated as a result of cold treatment [81]. Expression of cold induced proteins like β-phosphoglucomutase (β-Pgm), phospo acetyltransferase (Pat) and stress protection protein like UspA, after GSH supplementation helps to survive the cells during cold conditions. Over expression of GSH peroxidase counters the secondary oxidative stress generated as a result of cold treatment [81]. Exogenously added glutathione clearly protects the cells against various cold stress conditions but role of indigenously synthesized glutathione in this regard is yet to be explored.

### Osmotic stress

Resistance to osmotic stress is an important parameter for the industrial processing of microorganisms. Glutathione is already known to impart resistance against high osmotic conditions in *E. coli*[54,82]. Zhang and coworkers tested this role of GSH in osmoadaptation of *Lc. lactis* which could resist upto 5 M NaCl upon GSH supplementation [83]. Further, to infer the mechanism of GSH mediated osmotic protection, they followed a comparative proteomic approach which revealed upregulation of several glycolytic enzymes in GSH-supplemented cells during osmotic challenge. As microbial survival in high osmotic conditions is an energy intensive affair, upregulation of glycolytic pathway in LAB compensates for the additional energy expenditure. Additionally, the expression of proteins involved in the metabolism of other sugars was downregulated giving the bacteria a selective advantage for preferential utilization of simple sugars and thus to shut down the redundant metabolic processes. Moreover, GSH supplementation also increased the expression of certain stress resistance proteins [83]. The ability of supplemented GSH to protect against adverse osmotic conditions could be harnessed in cheese industry, where salting of curd blocks impart high osmotic stress to the starter bacteria and thus addition of glutathione to cheese milk or use of GSH accumulating or producing starters could offer protection resulting in shorter ripening time.

### Glutathione and aerobic respiration in LAB

Many LAB undergo aerobic respiration which have important technological ramifications for their use in industrial processing [84]. Respiration of LAB requires presence of *cydABCD* set of genes. *cydAB* encodes for structural components of a cytochrome oxidase known as the quinol oxidase. *cydDC* complex, on the other hand is required for the assembly of the cytochrome. As mentioned earlier, CydDC is also responsible for the transport of cysteine and glutathione, which contribute towards the reducing environment in the cell facilitating CydAB-heme interactions. Thus, presence of *cydDC* in LAB is essential for aerobic respiration in many species [85] and most of these species are also capable of transporting glutathione [see Additional file 1 and section 3.0]. However, there seems to be no correlation of aerobic respiration and glutathione biosynthesis ability in LAB.

## Technological implications of glutathione metabolism by LAB

Metabolism of glutathione by LAB has important physico-chemical implications for fermented foods. Redox reactions carried out by thiol compounds in sourdough considerably affects its rheology. *Lb*. *sanfranciscensis* has been known to be the most predominant bacterium in sourdough fermentation and its genomic analysis has revealed a well adapted cellular machinery for sourdough microenvironment [86]. Glutathione reductase activity of *Lb. sanfranciscensis* recycles GSSG to GSH in wheat sourdough and thus maintains a high reduced thiol concentration [69]. Glutathione is one of the most active thiol compound acting as a reducing agent and it reacts with the disulfide bonds in wheat proteins, thus restricting the disulphide mediated polymerization of gluten proteins in dough. This interference renders gluten peptides in depolymerized state, making them more water soluble which facilitates their proteolytic cleavage and thus imparting the required dough rheology [87]. Glutathione reductase activity and consequent thiol accumulation also result in egg white (EW) protein degradation in wheat sourdough. Structural changes in ovotransferrin, the major egg white protein, as result of reaction with thiols makes it more susceptible to proteolysis [88]. EW proteins are known to have certain biological activities influencing both technological and nutritional parameters of the processed food products and are also sought as the major structural proteins in the production of low gluten products [89].

Cheese production essentially involves ripening process to develop the desired delicate flavor and texture which requires controlled storage of green cheese blocks for a considerable period of time, increasing the cost of production and blocking the capital invested. Thus, various efforts to accelerate the process of ripening have been undertaken. Supplemented GSH has been reported to hasten the ripening process [90] but the cellular reactions describing the role have not been ascertained. Expression studies to assess the role of GSH system genes from LAB in cheese ripening process are still lacking. This role of glutathione may be attributed to its ability to enhance the metabolic activities in cheese starters as suggested by higher microbial esterase [91] and lipase [92] activities which directly relate to the flavor development and hence accelerate the process of ripening. GSH also acts as sulfur and nutrient source, thus promotes the growth of various LAB [40,43]. Moreover, GSH also helps maintain a low redox potential favorable for ripening process [18]. Addition of GSH increases the production of H_2_S and methanethiol, both important contributors to the overall cheese flavor [93]. The enzyme γ-glutamyl transpeptidase (Ggt), an enzyme capable of degrading GSH catalyzes the transfer of gamma glutamyl moiety of GSH, exposing the –SH group on the cysteine for H_2_S production. Raw milk exhibits GGT activity which is lost as a result of heat treatment and thus raw milk cheeses have more pronounced flavor. GGT activity is in general absent in LAB [19] which was also confirmed in our analysis by the absence of *ggt* homologs from the sequenced LAB genomes [see Additional file 1. Protection offered by GSH against high osmotic, acid and cold stress conditions, which would result in higher viability of metabolically active starter bacteria in cheese blocks, may also be a complementary factor in early ripening.

Commercial starter preparations require processing treatments which load the cells with varying stress conditions. For better survival and industrial performance of the cultures, they need to be resistant to such conditions. Given the multi stress tolerance achieved in LAB by GSH supplementation, it can be an important molecule for developing robust probiotic cultures with the ability to withstand harsh processing treatments as well as hostile conditions in the gut. It should also serve to promote the growth of starter bacteria and deliver added functionalities like flavor generation. The ability of GSH to act as a sulfur and cysteine source promotes the growth of *Oenococcus oeni* and malolactic fermentation in wine [94]. Glutathione reductase is present in most of the *Leu. mesenteroides* strains [95] and contributes to the sensory characteristics of wine by participating in the production of volatile sulfur compounds.

## Modulation of host antioxidative system: possible role of microbial glutathione metabolism

Glutathione has a widespread role in maintaining human health and upkeep of the immune system. Low glutathione level in humans is linked with a number of disease states such as cancer, AIDS, Alzheimer’s disease, Parkinson’s disease etc. [9]. Low levels of colonic glutathione and glutathione S-transferase activity are associated with a high risk for development of colorectal cancer [96]. The gut GSH system forms an essential antitoxic barrier for mucosa and helps maintain the normal immune functions [97]. Oxidative stress leading to an altered redox status of mucosal glutathione is a major etiological factor in ulcerative colitis, an inflammatory bowel disease (IBD) [98]. Thus for proper functioning, intestinal epithelial cells require a continuous supply of GSH. Exogenously supplied GSH was shown not only to maintain the required level in the gut but also offered protection against oxidative agents like t-butyl hydroperoxide or menadione in rat model [99].

Human gut is a hotspot for dynamic exchange reactions between commensal microorganisms and host tissues. Gut microbiota and probiotics impact the oxidative status of intestine [100] and the microbial antioxidant systems partly contribute towards this effect [101]. In recent years, probiotic bacteria targeted at mitigating oxidative stress induced damage have been tested for their beneficial effects to host. Antioxidative property of probiotic *Lb. fermentum* ME-3, besides other factors, is partly credited to its ability to produce (or accumulate) GSH and maintain high ratio of GSH/GSSG [37]. The strain is also reported to reduce the oxidative stress markers in blood and urine in humans [102]. *Lb. fermentum* 5716, a probiotic strain, is capable of ameliorating colonic inflammation in TNBS induced rat colitis which is also attributed to its ability to release glutathione in the gut [39]. *Lb. salivarius* CECT5713 could also show the same effect [103] but the microbial release of GSH was not tested, however *Lb. salivarius* may have the ability to release GSH [40]. *Lb. fermentum* was able to restore colonic GSH levels in a rat colitis model but *Lb. reuteri* lacked the ability and this effect could be correlated with the presumed GSH synthesis or high GSH accumulation capability in the former but absent in the latter [104].

Some reports have also suggested the promotion of host glutathione synthesis by probiotic bacteria but the exact contributing factors are not known. Human baby flora transinoculated in germ free mice resulted in an increased GSH biosynthesis in jejunum [105]. Three lactobacillus strains viz. *Lb. casei* CU001, *Lb. acidophilus* NCFM and *Lb. casei* YIT9018 were able to increase the systemic levels of GSH in mice and IgM production in spleen [106]. Similarly, a multispecies probiotic preparation induced upregulation of γ-glutamylcysteine ligase (GCL) activity and a consequent increase in the synthesis of GSH in the rat ileum. [41]. Probiotic mediated modulation of host GSH system is helpful in ameliorating disease conditions like acute pancreatitis [41] and lead induced oxidative stress [107]. Spyropoulos et.al. [108] also suggested the possible role of probiotic bacteria, having ability to either deliver GSH or promote host GSH synthesis, in treatment of radiation induced enteritis and colitis. Thus, a empirically designed probiotic intervention could be useful for treatments and conditions which are known to rapidly decrease mucosal glutathione levels like radiation therapy [109], *Helicobacter pylori* infection [110] etc.

## Conclusion

Once considered rare in Gram positive bacteria, glutathione is increasingly gaining attention in these organisms both as a naturally synthesized metabolite and more importantly as a media supplement. *S. thermophilus* and *E. faecalis* synthesize glutathione by the bifunctional fusion protein (GshF). Some other LAB like *Lb. plantarum*, *Lb. casei*, *Lb. rhamnosus*, *Lb. sakei* and *Lb. ruminis* also possess the fusion protein but GSH synthesis has not been established in these organisms. Most of the other species lack synthesis but are able to import it from the medium. Ability to import GSH has important physiological and technological implications for LAB. Supplementation studies have conclusively established its role in protection of LAB against many facets of stress. The exact mechanism of resistance offered and the systemic implications of glutathione supplementation need to be sought out but most of the reactions are supposed to be a manifestation of its antioxidative property. A logical and interesting extension would be to see if the natural producer strains are inherently robust to stressful conditions which are better tolerated by GSH supplementation. Such GSH producing strains can be used as adjunct cultures for accelerated ripening of cheese, improved sourdough fermentation and food grade GSH production. GSH can play a vital role in the development of robust probiotics for its role in stress tolerance. Health application of GSH producing or accumulating cultures is another exciting area but will require extensive experimental validation in different animal models. Milk (esp. whey proteins) serves all the necessary precursors for GSH synthesis and LAB having potential for glutathione biosynthesis could be used successfully for delivering this vital molecule in the human system through a milk or whey based functional fermented food. With its diverse role and immense benefits, glutathione can add a new dimension to the technological and health applications of lactic acid bacteria.

## Abbreviations

LAB: Lactic acid bacteria; GSH: Reduced glutathione; GSSG: Oxidized glutathione; ROS: Reactive oxygen species; CDM: Chemically defined medium; gshA: γ-glutamylcystiene synthetase; gshB: glutathione synthetase; gshF/gshAB: glutathione biosynthesis bifunctional fusion gene; *gor*/*gshR*: glutathione reductase; gpo: glutathione peroxidase; gst: glutathione S-transferase; cydDC: putative cysteine and glutathione importer.

## Competing interests

The authors declare that they have no competing interests.

## Authors’ contribution

SDP1 conceived the work and wrote the manuscript. SDP1 and SDP2 did the genomic analysis. SKT, RS and JK provided technical suggestions and reviewed the manuscript. All the authors have read and approved the manuscript before submission.

## Supplementary Material

Additional file 1 **Screening of selected LAB genomes for major genes of glutathione metabolism (synthesis, transport, redox turnover and degradation genes).** The numbers in cells indicate the number of genes found and the entries in bracket are uniprot ids (for Annotated genes) and (GI ids for RPS-Blast results). NA –Annotation not available in Uniprot. (XLS 73 kb) Click here for file

## References

[B1] van de Guchte M Serror P Chervaux C Smokvina T Ehrlich SD Maguin E Stress responses in lactic acid bacteria A Van Leeuw 2002 82 187 216 10.1023/A:1020631532202 12369188

[B2] Mills S Stanton C Fitzgerald GF Ross RP Enhancing the stress responses of probiotics for a lifestyle from gut to product and back again Microb Cell Fact 2011 10 Suppl 1 S19 10.1186/1475-2859-10-S1-S19 21995734 PMC3231925

[B3] Copley SD Dhillon JK Lateral gene transfer and parallel evolution in the history of glutathione biosynthesis genes Genome Biol 2002 3 1 16 10.1186/gb-2002-3-5-research0025 PMC115227 12049666

[B4] Fahey RC Brown WC Adams WB Worsham MB Occurrence of glutathione in bacteria J Bacteriol 1978 133 1126 1129 417060 10.1128/jb.133.3.1126-1129.1978 PMC222142

[B5] Gopal S Borovok I Ofer A Yanku M Cohen G Goebel W Kreft J Aharonowitz Y A multidomain fusion protein in Listeria monocytogenes catalyzes the two primary activities for glutathione biosynthesis J Bacteriol 2005 187 3839 3847 10.1128/JB.187.11.3839-3847.2005 15901709 PMC1112035

[B6] Janowiak BE Griffith OW Glutathione synthesis in Streptococcus agalactiae J Biol Chem 2005 280 11829 11839 10.1074/jbc.M414326200 15642737

[B7] Pittman MS Robinson HC Poole RK A bacterial glutathione transporter (Escherichia coli CydDC) exports reductant to the periplasm J Biol Chem 2005 280 32254 32261 10.1074/jbc.M503075200 16040611

[B8] Veeravalli K Boyd D Iverson BL Beckwith J Georgiou G Laboratory evolution of glutathione biosynthesis reveals natural compensatory pathways Nat Chem Biol 2011 7 101 105 21186348 10.1038/nchembio.499 PMC4129951

[B9] Wu G Fang YZ Yang S Lupton JR Turner ND Glutathione metabolism and its implications for health J Nutr 2004 134 489 492 14988435 10.1093/jn/134.3.489

[B10] Jones DP Redox potential of GSH/GSSG couple: Assay and biological significance Methods Enzymol 2002 348 93 112 11885298 10.1016/s0076-6879(02)48630-2

[B11] Schafer FQ Buettner GR Redox environment of the cell as viewed through the redox state of the glutathione disulfide/glutathione couple Free Radical Bio Med 2001 30 1191 1212 10.1016/S0891-5849(01)00480-4 11368918

[B12] Masip L Veeravalli K Georgiou G The many faces of glutathione in bacteria Antioxid Redox Sign 2006 8 753 762 10.1089/ars.2006.8.753 16771667

[B13] Smirnova GV Oktyabrsky ON Glutathione in bacteria Biochemistry (Moscow) 2005 70 1199 1211 10.1007/s10541-005-0248-3 16336178

[B14] Zhang WY Yu DL Sun ZH Airideng C Hu SN Meng H Zhang HP Preliminary analysis of glutathione S-transferase homolog from Lactobacillus casei Zhang Ann Microbiol 2009 59 727 731 10.1007/BF03179215

[B15] National Center for Biotechnology Information ftp://ftp.ncbi.nih.gov/genomes/Bacteria

[B16] Universal Protein Resource (Uniprot) http://www.uniprot.org

[B17] Klimke W Agarwala R Badretdin A Chetvernin S Ciufo S Fedorov B Kiryutin B O'Neill K Resch W Resenchuk S Schafer S Tolstoy L Ratusova T The national center for biotechnology information's protein clusters database Nucleic Acids Res 2009 37 D216 D223 http://www.ncbi.nlm.nih.gov/proteinclusters 10.1093/nar/gkn734 18940865 PMC2686591

[B18] Fernándes L Steele JL Glutathione content of lactic acid bacteria J Dairy Sci 1993 76 1233 1242 10.3168/jds.S0022-0302(93)77452-4

[B19] Wiederholt KM Steele JL Glutathione accumulation in lactococci J Dairy Sci 1994 77 1183 1188 10.3168/jds.S0022-0302(94)77056-9

[B20] Li Y Hugenholtz J Abee T Molenaar D Glutathione protects Lactococcus lactis against oxidative stress Appl Environ Microb 2003 69 5739 5745 10.1128/AEM.69.10.5739-5745.2003 PMC201183 14532020

[B21] Amaretti A di Nunzio M Pompei A Raimondi S Rossi M Bordoni A Antioxidant properties of potentially probiotic bacteria: in vitro and in vivo activities Appl Microbiol Biotin press 10.1007/s00253-012-4241-7 22790540

[B22] Musenga A Mandrioli R Bonifazi P Kenndler E Pompei A Raggi MA Sensitive and selective determination of glutathione in probiotic bacteria by capillary electrophoresis–laser induced fluorescence Anal Bioanal Chem 2007 387 917 924 10.1007/s00216-006-0980-6 17203251

[B23] Li Y Hugenholtz J Sybesma W Abee T Molenaar D Using Lactococcus lactis for glutathione overproduction Appl Microbiol Biot 2005 67 83 90 10.1007/s00253-004-1762-8 15490155

[B24] Xiang H Wei W Tan H Food-grade expression of human glutathione s-transferase and Cu/Zn superoxide dismutase in Lactococcus lactis Biomol Eng 2003 20 107 112 10.1016/S1389-0344(03)00007-8 12684072

[B25] Kim EK Cha CJ Cho YJ Cho YB Roe JH Synthesis of gamma-glutamylcysteine as a major low-molecular-weight thiol in lactic acid bacteria Leuconostoc spp Biochem Bioph Res Co 2008 369 1047 1051 10.1016/j.bbrc.2008.02.139 18329377

[B26] Liedschulte V Wachter A Zhigang A Rausch T Exploiting plants for glutathione (GSH) production: uncoupling GSH synthesis from cellular controls results in unprecedented GSH accumulation Plant biotechnol 2010 8 807 820 10.1111/j.1467-7652.2010.00510.x 20233332

[B27] Li W Li Z Yang J Ye Q Production of glutathione using a bifunctional enzyme encoded by gshF from Streptococcus thermophilus expressed in Escherichia coli J Biotechnol 2011 154 261 268 10.1016/j.jbiotec.2011.06.001 21683099

[B28] Newton GL Arnold K Price MS Sherrill C Delcardayre SB Aharonowitz Y Cohen G Davies J Fahey RC Davis C Distribution of thiols in microorganisms: mycothiol is a major thiol in most actinomycetes J Bacteriol 1996 178 1990 1995 8606174 10.1128/jb.178.7.1990-1995.1996 PMC177895

[B29] Pebay M Holl AC Simonet JM Decaris B Characterization of the gor gene of the lactic acid bacterium Streptococcus thermophilus CNRZ368 Res Microbiol 1995 146 371 383 10.1016/0923-2508(96)80283-X 8525054

[B30] Goh YJ Goin C O’Flaherty S Altermann E Hutkins R Specialized adaptation of a lactic acid bacterium to the milk environment: the comparative genomics of Streptococcus thermophilus LMD-9 Microb Cell Fact 2011 10 Suppl 1 S22 10.1186/1475-2859-10-S1-S22 21995282 PMC3231929

[B31] Lo´pez G Latorre M Reyes-Jara A Cambiazo V Gonza´lez M Transcriptomic response of Enterococcus faecalis to iron excess Biometals 2012 25 737 747 10.1007/s10534-012-9539-5 22447126

[B32] Griffith OW Janowiak BE Bifunctional enzyme with γ-glutamylcysteine synthetase and glutathione synthetase activity and uses thereof 2008 Patent 0194701 A1, 2008, United States 14-08-2008

[B33] Patel MP Marcinkeviciene J Blanchard JS Enterococcus faecalis glutathione reductase: purification, characterization and expression under normal and hyperbaric O2 conditions FEMS Microbiol Lett 1998 166 155 163 10.1111/j.1574-6968.1998.tb13197.x 9741094

[B34] Yoon YH Byun JR Occurrence of glutathione sulphydryl (GSH) and antioxidant activities in probiotic Lactobacillus spp Asian-Aust J Anim Sci 2004 17 1582 1585

[B35] Serata M Iino T Yasuda E Sako T Roles of thioredoxin and thioredoxin reductase in the resistance to oxidative stress in Lactobacillus casei Microbiology 2012 158 953 962 10.1099/mic.0.053942-0 22301908

[B36] Kullisaar T Songisepp E Aunapuu M Kilk K Arend A Mikelsaar M Rehema A Zilmer M Complete glutathione system in probiotic Lactobacillus fermentum ME-3 Appl Biochem Micro 2010 46 481 486 10.1134/S0003683810050030 21058502

[B37] Kullisaar T Zilmer M Mikelsaar M Vihalemm T Annuk H Kairane C Kilk A Two antioxidative lactobacilli strains as promising probiotics Int J Food Microbiol 2002 72 215 224 10.1016/S0168-1605(01)00674-2 11845820

[B38] Mikelsaar M Zilmer M Lactobacillus fermentum ME-3-an antimicrobial and antioxidative probiotic Microb Ecol Health D 2009 21 1 27 10.1080/08910600902815561 PMC2670518 19381356

[B39] Peran L Camuesco D Comalada M Nieto A Concha A Adrio JL Olivares M Xaus J Zarzuelo A Galvez J Lactobacillus fermentum, a probiotic capable to release glutathione, prevents colonic inflammation in the TNBS model of rat colitis Int J Colorectal Dis 2006 21 737 746 10.1007/s00384-005-0773-y 16052308

[B40] Lee KB Pi KB Kim EB Rho BS Kang SK Lee HG Choi YJ Glutathione-mediated response to acid stress in the probiotic bacterium, Lactobacillus salivarius Biotechnol Lett 2010 32 969 972 10.1007/s10529-010-0244-6 20349113

[B41] Lutgendorff F Nijmeijer RM Sandström PA Trulsson LM Magnusson KE Timmerman HM Van Minnen LP Rijkers GT Gooszen HG Akkermans LMA Probiotics prevent intestinal barrier dysfunction in acute pancreatitis in rats via induction of ileal mucosal glutathione biosynthesis PLoS One 2009 4 e4512 10.1371/journal.pone.0004512 19223985 PMC2639703

[B42] Lo R Turner MS Barry DG Sreekumar R Walsh TP Giffard PM Cystathionine γ-lyase is a component of cystine-mediated oxidative defense in Lactobacillus reuteri BR11 J Bacteriol 2009 191 1827 1837 10.1128/JB.01553-08 19124577 PMC2648363

[B43] Lee KB Kim HJ Rho BS Kang SK Choi YJ Effect of glutathione on growth of the probiotic bacterium Lactobacillus reuteri Biochemistry (Moscow) 2011 76 423 426 10.1134/S0006297911040043 21585317

[B44] Ge S Zhu T Li Y Expressing bacterial GshF in Pichia pastoris for glutathione production Appl Environ Microb 2012 78 5435 5439 10.1128/AEM.00509-12 PMC3416392 22610434

[B45] Serrano LM Molenaar D Wels M Teusink B Bron P de Vos W Smid E Thioredoxin reductase is a key factor in the oxidative stress response of Lactobacillus plantarum WCFS1 Microb Cell Fact 2007 6 29 10.1186/1475-2859-6-29 17725816 PMC2174512

[B46] Guilbaud M Zagorec M Chaillou S Champomier-Vergès MC Intraspecies diversity of Lactobacillus sakei response to oxidative stress and variability of strain performance in mixed strains challenges Food Microbiol 2012 29 197 204 10.1016/j.fm.2011.07.011 22202873

[B47] Forde BM Neville BA O’Donnell MM Riboulet-Bisson E Claesson MJ Coghlan A Ross RP O’Toole PW Genome sequences and comparative genomics of two Lactobacillus ruminis strains from the bovine and human intestinal tracts Microb Cell Fact 2011 10 Suppl 1 S13 10.1186/1475-2859-10-S1-S13 21995554 PMC3231920

[B48] Allocati N Federici L Masulli M Di Ilio C Distribution of glutathione transferases in Gram-positive bacteria and Archaea Biochimie 2012 94 588 596 10.1016/j.biochi.2011.09.008 21945597

[B49] Falentin H Deutsch SM Jan G Loux V Thierry A Parayre S Maillard MB Dherbécourt J Cousin FJ Jardin J The complete genome of Propionibacterium freudenreichii CIRM-BIA1T, a hardy actinobacterium with food and probiotic applications PLoS One 2010 5 529 546 10.1371/journal.pone.0011748 PMC2909200 20668525

[B50] Vorobjeva LI Khodjaev EY Cherdinceva TA The study of induced antimutagenesis of propionic acid bacteria J Microbiol Meth 1996 24 249 258 10.1016/0167-7012(95)00044-5

[B51] Carmel-Harel O Storz G Roles of the glutathione-and thioredoxin-dependent reduction systems in the Escherichia coli and Saccharomyces cerevisiae responses to oxidative stress Annu Rev Microbiol 2000 54 439 461 10.1146/annurev.micro.54.1.439 11018134

[B52] Moore WR Anderson ME Meister A Murata K Kimura A Increased capacity for glutathione synthesis enhances resistance to radiation in Escherichia coli: a possible model for mammalian cell protection P Natl Acad Sci USA 1989 86 1461 1464 10.1073/pnas.86.5.1461 PMC286716 2564202

[B53] Ferguson GP Booth IR Importance of glutathione for growth and survival of Escherichia coli cells: detoxification of methylglyoxal and maintenance of intracellular K+ J Bacteriol 1998 180 4314 4318 9696786 10.1128/jb.180.16.4314-4318.1998 PMC107434

[B54] Smirnova GV Krasnykh TA Oktyabrsky ON Role of glutathione in the response of Escherichia coli to osmotic stress Biochemistry (Moscow) 2001 66 973 978 10.1023/A:1012361323992 11703177

[B55] Chesney JA Eaton JW Mahoney JR Jr Bacterial glutathione: a sacrificial defense against chlorine compounds J Bacteriol 1996 178 2131 2135 8606194 10.1128/jb.178.7.2131-2135.1996 PMC177915

[B56] Helbig K Bleuel C Krauss GJ Nies DH Glutathione and transition-metal homeostasis in Escherichia coli J Bacteriol 2008 190 5431 5438 10.1128/JB.00271-08 18539744 PMC2493246

[B57] Brenot A King KY Janowiak B Griffith O Caparon MG Contribution of glutathione peroxidase to the virulence of Streptococcus pyogenes Infect Immun 2004 72 408 413 10.1128/IAI.72.1.408-413.2004 14688122 PMC344014

[B58] Mishra S Imlay J Why do bacteria use so many enzymes to scavenge hydrogen peroxide? Arch Biochem Biophys 2012 525 145 160 10.1016/j.abb.2012.04.014 22609271 PMC3413786

[B59] Vido K Diemer H Van Dorsselaer A Leize E Juillard V Gruss A Gaudu P Roles of thioredoxin reductase during the aerobic life of Lactococcus lactis J Bacteriol 2005 187 601 610 10.1128/JB.187.2.601-610.2005 15629931 PMC543548

[B60] Hung J Cooper D Turner MS Walsh T Giffard PM Cystine uptake prevents production of hydrogen peroxide by Lactobacillus fermentum BR11 FEMS Microbiol Lett 2003 227 93 99 10.1016/S0378-1097(03)00653-0 14568153

[B61] Oktyabrskii ON Smirnova GV Redox potential changes in bacterial cultures under stress conditions Microbiology 2012 81 131 142 10.1134/S0026261712020099

[B62] Michelon D Abraham S Ebel B De Coninck J Husson F Feron G Gervais P Cachon R Contribution of exofacial thiol groups in the reducing activity of Lactococcus lactis FEBS J 2010 277 2282 2290 10.1111/j.1742-4658.2010.07644.x 20423456

[B63] Grant CM MacIver FH Dawes IW Glutathione is an essential metabolite required for resistance to oxidative stress in the yeast Saccharomyces cerevisiae Curr Genet 1996 29 511 515 10.1007/BF02426954 8662189

[B64] Vergauwen B Pauwels F Vaneechoutte M Van Beeumen JJ Exogenous glutathione completes the defense against oxidative stress in Haemophilus influenzae J Bacteriol 2003 185 1572 1581 10.1128/JB.185.5.1572-1581.2003 12591874 PMC148052

[B65] Sherrill C Fahey RC Import and metabolism of glutathione by Streptococcus mutans J Bacteriol 1998 180 1454 1459 9515913 10.1128/jb.180.6.1454-1459.1998 PMC107044

[B66] Fu RY Bongers RS Van Swam II Chen J Molenaar D Kleerebezem M Hugenholtz J Li Y Introducing glutathione biosynthetic capability into Lactococcus lactis subsp. cremoris NZ9000 improves the oxidative-stress resistance of the host Metab Eng 2006 8 662 671 10.1016/j.ymben.2006.07.004 16962352

[B67] Zhu L Dong H Zhang Y Li Y Engineering the robustness of Clostridium acetobutylicum by introducing glutathione biosynthetic capability Metab Eng 2011 13 426 434 10.1016/j.ymben.2011.01.009 21296183

[B68] Kim HS Chae HS Jeong SG Ham JS Im SK Ahn CN Lee JM In vitro antioxidative properties of lactobacilli Asian-Aust J Anim Sci 2006 19 262 265

[B69] Jansch A Korakli M Vogel RF Gänzle MG Glutathione reductase from Lactobacillus sanfranciscensis DSM20451T: contribution to oxygen tolerance and thiol exchange reactions in wheat sourdoughs Appl Environ Microbiol 2007 73 4469 4476 10.1128/AEM.02322-06 17496130 PMC1932818

[B70] Girgis HS Cano RJ Klaenhammer TR Tolerance to hydrogen peroxide and expression of glutathione reductase in Lactobacillus Annual meeting, Institute of Food Technologists 10–14 June 2000 2000, Dallas, Texas 78D–10

[B71] Bron PA Molenaar D Vos WM Kleerebezem M DNA micro array based identification of bile responsive genes in Lactobacillus plantarum J Appl Microbiol 2006 100 728 738 10.1111/j.1365-2672.2006.02891.x 16553727

[B72] Dressaire C Redon E Gitton C Loubière P Monnet V Cocaign-Bousquet M Investigation of the adaptation of Lactococcus lactis to isoleucine starvation integrating dynamic transcriptome and proteome information Microb Cell Fact 2011 10 Suppl 1 S18 10.1186/1475-2859-10-S1-S18 21995707 PMC3236307

[B73] Meury J Kepes A Glutathione and the gated potassium channels of Escherichia coli EMBO J 1982 1 339 343 6325160 10.1002/j.1460-2075.1982.tb01171.x PMC553046

[B74] Riccillo PM Muglia CI De Bruijn FJ Roe AJ Booth IR Aguilar OM Glutathione is involved in environmental stress responses in Rhizobium tropici, including acid tolerance J Bacteriol 2000 182 1748 1753 10.1128/JB.182.6.1748-1753.2000 10692382 PMC94474

[B75] Zhang J Fu RY Hugenholtz J Li Y Chen J Glutathione protects Lactococcus lactis against acid stress Appl Environ Microbiol 2007 73 5268 5275 10.1128/AEM.02787-06 17601814 PMC1950978

[B76] Kim JE Eom HJ Kim Y Ahn JE Kim JH Han NS Enhancing acid tolerance of Leuconostoc mesenteroides with glutathione Biotechnol Lett 2012 34 683 687 10.1007/s10529-011-0815-1 22160366

[B77] Xie Y Chou L Cutler A Weimer B DNA macroarray profiling of Lactococcus lactis subsp. lactis IL1403 gene expression during environmental stresses Appl Environ Microbiol 2004 70 6738 6747 10.1128/AEM.70.11.6738-6747.2004 15528540 PMC525116

[B78] Martin-Galiano AJ Overweg K Ferrandiz MJ Reuter M Wells JM De la Campa AG Transcriptional analysis of the acid tolerance response in Streptococcus pneumoniae Microbiology 2005 151 3935 3946 10.1099/mic.0.28238-0 16339938

[B79] Smirnova GV Zakirova ON Oktyabrsky ON Role of the antioxidant system in response of Escherichia coli bacteria to cold stress Mikrobiologiia 2001 70 55 60 11338838

[B80] Zhang J Du GC Zhang Y Liao XY Wang M Li Y Chen J Glutathione protects Lactobacillus sanfranciscensis against freeze-thawing, freeze-drying, and cold treatment Appl Environ Microbiol 2010 76 2989 2996 10.1128/AEM.00026-09 20208023 PMC2863433

[B81] Zhang J Li Y Chen W Du GC Chen J Glutathione improves the cold resistance of Lactobacillus sanfranciscensis by physiological regulation Food Microbiol 2012 31 285 292 10.1016/j.fm.2012.04.006 22608235

[B82] McLaggan D Logan TM Lynn DG Epstein W Involvement of gamma-glutamyl peptides in osmoadaptation of Escherichia coli J Bacteriol 1990 172 3631 3636 1972940 10.1128/jb.172.7.3631-3636.1990 PMC213336

[B83] Zhang Y Zhu Y Mao S Li Y Proteomic analyses to reveal the protective role of glutathione in resistance of Lactococcus lactis to osmotic stress Appl Environ Microbiol 2010 76 3177 3186 10.1128/AEM.02942-09 20348298 PMC2869154

[B84] Pedersen MB Gaudu P Lechardeur D Petit MA Gruss A Respiration pathways in fermentative lactic acid bacteria and uses in biotechnology Ann Rev Food Sci Technol 2012 3 37 58 10.1146/annurev-food-022811-101255 22385163

[B85] Brooijmans R Smit B Santos F Van Riel J De Vos WM Hugenholtz J Heme and menaquinone induced electron transport in lactic acid bacteria Microb Cell Fact 2009 8 28 10.1186/1475-2859-8-28 19480672 PMC2696406

[B86] Rudi V Melanie P Matthias E Arnim W Heiko L Stefanie O Sonja V Angel A Georg B Wolfgang L Genomic analysis reveals Lactobacillus sanfranciscensis as stable element in traditional sourdoughs Microb Cell Fact 2011 10 Suppl 1 S6 10.1186/1475-2859-10-S1-S6 21995419 PMC3231932

[B87] Gänzle MG Loponen J Gobbetti M Proteolysis in sourdough fermentations: mechanisms and potential for improved bread quality Trends Food Sci Tech 2008 19 513 521 10.1016/j.tifs.2008.04.002

[B88] Loponen J König K Wu J Gänzle MG Influence of thiol metabolism of lactobacilli on egg white proteins in wheat sourdoughs J Agr Food Chem 2008 56 3357 3362 10.1021/jf703600t 18412365

[B89] Erem F Sontag-Strohm T Certel M Salovaara H Loponen J Functional characteristics of egg white proteins within wheat, rye, and germinated-rye sourdoughs J Agr Food Chem 2009 58 1263 1269 20043636 10.1021/jf903228x

[B90] Singh S Kristoffersen T Factors affecting flavor development in cheddar cheese slurries J Dairy Sci 1970 53 533 536 10.3168/jds.S0022-0302(70)86247-6

[B91] Harper WJ Carmona de Catril A Chen JL Esterases of lactic streptococci and their stability in cheese slurry systems Milchwissenschaft 1980 35 129 132

[B92] Kamaly KM Marth EH Enzyme activities of lactic streptococci and their role in maturation of cheese: a review J Dairy Sci 1989 72 1945 1966 10.3168/jds.S0022-0302(89)79318-8

[B93] Urbach G Contribution of lactic acid bacteria to flavour compound formation in dairy products Int Dairy J 1995 5 877 903 10.1016/0958-6946(95)00037-2

[B94] Rauhut D Gawron-Scibek M Beisert B Kondzior M Schwarz R Kürbel H Grossmann M Krieger S Impact of S-containing amino acids and glutathione on growth of Oenococcus oeni and malolactic fermentation LES XVIe Entretiens Scientifiques Lallemand: 4–5 May 2004 2004, Porto 33 38

[B95] Mtshali PS Divol B du Toit M PCR detection of enzyme-encoding genes in Leuconostoc mesenteroides strains of wine origin World J Microb Biot 2012 28 1443 1449 10.1007/s11274-011-0944-7 22805925

[B96] Grubben M Den Braak CCM Nagengast FM Peters WHM Low colonic glutathione detoxification capacity in patients at risk for colon cancer Eur J Clin Invest 2006 36 188 192 10.1111/j.1365-2362.2006.01618.x 16506964

[B97] Hoensch H Morgenstern I Petereit G Siepmann M Peters WHM Roelofs HMJ Kirch W Influence of clinical factors, diet, and drugs on the human upper gastrointestinal glutathione system Gut 2002 50 235 240 10.1136/gut.50.2.235 11788566 PMC1773114

[B98] Holmes EW Yong SL Eiznhamer D Keshavarzian A Glutathione content of colonic mucosa (Evidence for oxidative damage in active ulcerative colitis) Digest Dis Sci 1998 43 1088 1095 10.1023/A:1018899222258 9590426

[B99] Lash LH Hagen TM Jones DP Exogenous glutathione protects intestinal epithelial cells from oxidative injury P Natl Acad Sci USA 1986 83 4641 4645 10.1073/pnas.83.13.4641 PMC323797 3460063

[B100] Circu ML Aw TY Redox biology of the intestine Free Radical Res 2011 45 1245 1266 10.3109/10715762.2011.611509 21831010 PMC3210416

[B101] Ginsburg I Kohen R Koren E Microbial and host cells acquire enhanced oxidant-scavenging abilities by binding polyphenols Arch Biochem Biophys 2011 506 12 23 10.1016/j.abb.2010.11.009 21081104

[B102] Songisepp E Kals J Kullisaar T Mändar R Hutt P Zilmer M Mikelsaar M Evaluation of the functional efficacy of an antioxidative probiotic in healthy volunteers Nutr J 2005 4 22 10.1186/1475-2891-4-22 16080791 PMC1198254

[B103] Peran L Camuesco D Comalada M Nieto A Concha A Diaz-Ropero MP Olivares M Xaus J Zarzuelo A Galvez J Preventative effects of a probiotic, Lactobacillus salivarius ssp. salivarius, in the TNBS model of rat colitis World J Gastroentero 2005 11 5185 5192 10.3748/wjg.v11.i33.5185 PMC4320393 16127750

[B104] Peran L Sierra S Comalada M Lara-Villoslada F Bailón E Nieto A Concha A Olivares M Zarzuelo A Xaus J A comparative study of the preventative effects exerted by two probiotics, Lactobacillus reuteri and Lactobacillus fermentum, in the trinitrobenzenesulfonic acid model of rat colitis Brit J Nutr 2007 97 96 103 10.1017/S0007114507257770 17217564

[B105] Martin FPJ Wang Y Yap IKS Sprenger N Lindon JC Rezzi S Kochhar S Holmes E Nicholson JK Topographical variation in murine intestinal metabolic profiles in relation to microbiome speciation and functional ecological activity J Proteome Res 2009 8 3464 3474 10.1021/pr900099x 19492798

[B106] Byun JR Baik YJ Yoon YH Effects of feeding Lactobacillus spp. on the level of cell glutathione sulphydryl and immunoglobulin M in ICR mice Asian-Aust J Anim Sci 2004 17 415 419

[B107] Tian F Zhai Q Zhao J Liu X Wang G Zhang H Chen W Lactobacillus plantarum CCFM8661 alleviates lead toxicity in mice Biol Trace Elem Resin press 10.1007/s12011-012-9462-1 22684513

[B108] Spyropoulos BG Misiakos EP Fotiadis C Stoidis CN Antioxidant properties of probiotics and their protective effects in the pathogenesis of radiation-induced enteritis and colitis Digest Dis Sci 2011 56 285 294 10.1007/s10620-010-1307-1 20632107

[B109] Mutlu-Türkolu Ü Erbil Y Öztezcan S Olgaç V Token G Uysal M The effect of selenium and/or vitamin E treatments on radiation-induced intestinal injury in rats Life Sci 2000 66 1905 1913 10.1016/S0024-3205(00)00516-6 10821115

[B110] Shirin H Pinto JT Liu LU Merzianu M Sordillo EM Moss SF Helicobacter pylori decreases gastric mucosal glutathione Cancer Lett 2001 164 127 133 10.1016/S0304-3835(01)00383-4 11179826

[B111] Cleusix V Lacroix C Vollenweider S Duboux M Le Blay G Inhibitory activity spectrum of reuterin produced by Lactobacillus reuteri against intestinal bacteria BMC Microbiol 2007 7 101 10.1186/1471-2180-7-101 17997816 PMC2222629

